# Increased Liver Stiffness in Systemic Sclerosis: A Single-Centre Transient Elastography Study

**DOI:** 10.3390/jcm15218593

**Published:** 2026-11-04

**Authors:** Nicola Ortalli, Alberto Civolani, Simone Aldo Lari, Emanuela Vargiu, Erika Meleddu, Maria Maddalena Angioni, Alberto Floris, Sara Onali, Matteo Piga, Massimo Claudio Fantini, Alessandra Vacca, Alberto Cauli

**Affiliations:** 1 Department of Medical Sciences and Public Health, University of Cagliari, 09042 Monserrato, Italy; 2 Rheumatology Unit, Azienda Ospedaliero Universitaria di Cagliari, 09042 Monserrato, Italy; 3 Gastroenterology Unit, Azienda Ospedaliero Universitaria di Cagliari, 09042 Monserrato, Italy; 4 Rheumatology Unit, ASL Sulcis Iglesiente, 09012 Iglesias, Italy

**Keywords:** systemic sclerosis, liver stiffness, transient elastography, liver fibrosis, FibroScan

## Abstract

**Background/Objectives:** This study aimed to evaluate whether liver stiffness (LS), as assessed by transient elastography (TE), is increased in patients with systemic sclerosis (SSc) and to identify the factors associated with LS. **Methods:** In this exploratory cross-sectional study, the LS value and the prevalence of LS ≥ 5.3 and LS ≥ 7.5 kPa in 94 SSc patients and 50 healthy controls (HCs) were compared by univariate and multivariate analyses (adjusted for age and sex). TE was performed using FibroScan^®^ and LS values were expressed in kPa. Clinical and laboratory variables were analysed as factors potentially associated with LS, LS ≥ 5.3 kPa and LS ≥ 7.5 kPa, in exploratory analyses adjusted for a pre-specified set of confounders. **Results:** Among the 94 SSc patients, 79 (84%) were female; median age was 59 (49–70) years, and median disease duration was 8 (3–13) years. LS was significantly higher in SSc patients than in HCs in both univariate (5.12 ± 1.95 vs. 4.18 ± 0.98 kPa, *p* < 0.001) and multivariate analyses (β = 0.289, *p* = 0.001), with a higher prevalence of LS ≥ 5.3 kPa (40.4% vs. 10.0%, adjOR 6.85, *p* = 0.001) and LS ≥ 7.5 kPa (11.7% vs. 0%, *p* = 0.009). Only a limited number of factors were associated with LS abnormalities: telangiectasias (β = 0.222, *p* = 0.044) and glutamic–oxaloacetic transaminase (GOT) (β = 0.278, *p* = 0.012) for LS, and GOT (adjOR 1.082, *p* = 0.020) and anti-centromere antibodies (adjOR 4.09, *p* = 0.017) for LS ≥ 5.3 kPa. In univariate analysis, LS ≥ 7.5 kPa was associated with pulmonary hypertension (*p* = 0.049), pre-capillary pulmonary hypertension (*p* = 0.011), and GOT (*p* = 0.032); no multivariate model was fitted because of the few events. **Conclusions:** SSc patients show increased LS values and a higher prevalence of both LS ≥ 5.3 kPa and LS ≥ 7.5 kPa than HCs, easily detectable by TE. These findings are exploratory and require confirmation in larger cohorts to determine whether increased LS in SSc reflects hepatic fibrosis and whether it carries clinical and prognostic implications.

## 1. Introduction

Systemic sclerosis (SSc) is a complex autoimmune disease characterised by inflammation, progressive fibrosis of the skin and internal organs, and associated microvascular alterations [1]. The pathogenesis of SSc is defined by three main mechanisms: vascular loss and occlusive vasculopathy, immune cell activation and dysregulation, and excessive extracellular matrix deposition leading to fibrosis [2]. While the exact underlying mechanism remains unknown, it appears that vascular loss primarily stems from vascular dysfunction, which induces hypoxia, endothelial damage, and activation [3].

This, in turn, triggers an aberrant activation of the immune system, which contributes to the development and progression of fibrosis [4]. Fibrosis in SSc reflects a dysregulated repair process characterised by persistent fibroblast activation and excessive extracellular matrix deposition, leading to progressive tissue remodelling and organ dysfunction [2]. Major organ involvement in SSc, affecting the skin, heart, lungs, joints, muscles, and gastrointestinal tract, is well-described due to its significant clinical impact and burden on patients [5]. In contrast, liver alterations are more challenging to assess owing to the general lack of liver-specific symptoms. Biochemical liver abnormalities in SSc patients have been reported in the context of drug use, alcohol consumption, and coexisting infectious or autoimmune liver diseases [6]. Indeed, liver involvement in SSc is frequently observed in association with other hepatic conditions, such as autoimmune hepatitis or primary biliary cholangitis (PBC), which often co-occur with SSc [7,8,9].

The most frequent causes of chronic liver disease in the general population include infections from hepatotropic viruses (hepatitis B virus (HBV) or hepatitis C virus (HCV)), alcohol consumption, metabolic diseases such as metabolic dysfunction-associated steatotic liver disease (MASLD, formerly non-alcoholic fatty liver disease), autoimmune processes (autoimmune hepatitis or PBC), or hereditary conditions (e.g., hemochromatosis and Wilson disease) [10]. Hepatic fibrosis represents the natural consequence of chronic liver injury, where abnormal reparative processes lead to the accumulation of extracellular matrix, thereby altering the complex architecture of the hepatic parenchyma [11]. Liver fibrosis has been reported in a subset of SSc patients, though whether this reflects a direct pro-fibrotic effect of the disease or the coexistence of other hepatic conditions remains unclear [12]. Currently, hepatic biopsy is considered the gold standard for assessing liver fibrosis [13]. However, this method is an invasive procedure and carries risks of complications. Consequently, to assess the degree of liver fibrosis while minimising the need for biopsy, various non-invasive methods have been developed and proposed in recent years. These include transient elastography (TE) and blood tests (e.g., FibroTest^®^/FibroSure^®^, FibroMeter^®^), which have demonstrated good reliability when compared to the gold standard [14,15,16].

Previous studies, including recent elastography-based analyses, have provided insights into liver involvement in SSc; however, they often include patients with metabolic risk factors, biochemical abnormalities, or coexisting liver disease, limiting the ability to isolate the contribution of SSc itself.

This study aimed to evaluate whether liver stiffness (LS) is increased in patients with SSc and to identify the factors associated with it.

## 2. Patients and Methods

### 2.1. Patients and Study Design

In this cross-sectional study, 94 SSc patients who underwent TE at the Scleroderma Clinic of the University Hospital of Cagliari (Italy) were included, along with 50 healthy controls (HCs). Patients and HCs were enrolled between July 2013 and July 2023. During this period, all SSc patients attending the clinic were consecutively invited to participate; those who fulfilled the inclusion criteria and agreed to take part in the study were enrolled after signing written informed consent. Each patient underwent a single TE examination, and all clinical, serological, and laboratory data refer to the visit at which TE was performed. Inclusion criteria were (i) confirmed diagnosis of SSc; (ii) fulfilment of the ACR/EULAR 2013 classification criteria; (iii) age ≥ 18 years; and (iv) absence of known liver disease, alcohol abuse, or other conditions associated with liver fibrosis. To further exclude subclinical or unrecognised liver involvement, all patients underwent serological testing for hepatitis B virus, hepatitis C virus, anti-liver–kidney microsomal (LKM) antibodies, and anti-mitochondrial antibodies (AMAs).

HCs were recruited among healthcare personnel of the University Hospital of Cagliari over the same period. Inclusion criteria were age ≥ 18 years and the absence of known chronic diseases, including liver disease, alcohol abuse, and the use of potentially hepatotoxic drugs. As part of the mandatory occupational health surveillance, all HCs had documented negative serology for HBV and HCV; LKM and AMA antibodies were not tested in HCs. Since they were not matched with SSc patients for age and sex, the comparative analyses were adjusted for these demographic variables.

The study was approved by the Ethics Committee of the Azienda Ospedaliero-Universitaria di Cagliari on 24 June 2013 (Protocol No. NP/2013/3837). Written informed consent was obtained from all participants.

### 2.2. Clinical Data Collection

For all recruited SSc patients, demographic and anthropometric characteristics (age and sex, disease duration since the first non-Raynaud symptom, and body mass index), clinical features (limited or diffuse cutaneous SSc, interstitial lung disease, pulmonary hypertension, oesophageal involvement, history of digital ulcers, calcinosis, telangiectasias, and Raynaud’s phenomenon), serological profile (antinuclear antibodies, anti-Scl-70 antibodies, anticentromere antibodies, and other relevant autoantibodies), laboratory findings (glutamic–oxaloacetic transaminase [GOT], glutamic–pyruvic transaminase [GPT], gamma-glutamyl transferase [GGT], alkaline phosphatase [ALP], erythrocyte sedimentation rate [ESR], C-reactive protein [CRP], and total cholesterol), pulmonary function tests (forced vital capacity [FVC] and diffusing capacity for carbon monoxide [DLCO]), comorbidities (arterial hypertension, and diabetes), and treatment characteristics (immunosuppressive therapy, antifibrotic, methotrexate, history of corticosteroids, endothelin receptor antagonists, history of prostanoids, and phosphodiesterase-5 inhibitors) were recorded (Table 1). ILD was defined as interstitial abnormalities consistent with SSc-ILD on HRCT, as reported by the radiologist. All patients underwent annual screening for pulmonary hypertension with transthoracic echocardiography, pulmonary function tests with DLCO, and N-terminal pro-B-type natriuretic peptide (NT-proBNP); those with an intermediate or high echocardiographic probability were referred for right heart catheterisation (RHC). Twelve patients underwent RHC. Pulmonary hypertension (PH) was defined haemodynamically as mean pulmonary arterial pressure (mPAP) ≥ 25 mmHg and classified as pre-capillary (pulmonary artery wedge pressure [PAWP] ≤ 15 mmHg and pulmonary vascular resistance [PVR] > 3 Wood units) or post-capillary (PAWP > 15 mmHg).

Oesophageal involvement was defined by abnormalities documented on manometry, endoscopy, or barium swallow. Digital ulcers were recorded if present at the time of TE or at any time before it.

### 2.3. Transient Elastography Examination

To evaluate the LS, all enrolled subjects and HCs underwent TE using a FibroScan^®^ 502 device (Echosens, Paris, France) equipped with the M probe. The device was not equipped with the controlled attenuation parameter (CAP) software module, and CAP values were therefore not available. TE is not part of the routine diagnostic work-up of SSc at our centre; all examinations were performed specifically for the purposes of this study. All examinations were performed in the fasting state. The examination was performed by a trained gastroenterologist, who was blinded to the patients’ medical conditions and blood sample analyses, in accordance with the EASL-ALEH 2015 guidelines [17]. The examination was performed on the right hepatic lobe, through intercostal spaces, with the patient in a supine position and the right arm in maximal abduction. A TE examination was considered valid if at least 10 successful measurements were obtained, with a success rate ≥ 60%, and an interquartile range-to-median ratio (IQR/median) ≤ 30%. Liver stiffness values are expressed in kilopascals (kPa). Based on previously reported LS values in TE studies [18,19,20], two thresholds were exploratorily defined: LS ≥ 5.3 kPa for the primary analysis and a higher threshold of LS ≥ 7.5 kPa for the secondary analysis.

### 2.4. Statistical Analysis

Normality of continuous variables was assessed using the Shapiro–Wilk test. Normally distributed variables are presented as mean (standard deviation, SD) and compared using Student’s *t*-test; non-normally distributed variables are presented as median (interquartile range, IQR) and compared using the Mann–Whitney U test. Categorical variables are presented as numbers and percentages and compared using Fisher’s exact test or the chi-square test, as appropriate. Correlations between liver stiffness and continuous variables were assessed using Spearman’s rank correlation coefficient, chosen over Pearson’s correlation because most variables were not normally distributed.

LS values in SSc patients and HCs were compared by linear regression in multivariate analysis (results expressed as beta coefficient, β). The prevalence of LS ≥ 5.3 and of LS ≥ 7.5 kPa in SSc patients versus HCs was compared by Fisher’s exact test in univariate analysis and by logistic regression in multivariate analysis (results expressed as adjusted odds ratios, adjOR, with 95% confidence intervals). Comparisons between SSc patients and HCs were adjusted for age and sex, as the two groups were not matched for these variables.

Within the SSc cohort, the factors associated with liver stiffness were examined by comparing LS values between patients with and without each dichotomous clinical feature, and by correlating LS with continuous variables. Covariates for the multivariate models were selected a priori on the basis of established determinants of liver stiffness and comprised age, sex, BMI, total cholesterol, pulmonary hypertension, and methotrexate therapy. The same covariate set was used in all models. Because of the small number of patients with LS ≥ 7.5 kPa, no multivariate model was fitted for this outcome. Missing data were not imputed. Each analysis included only participants with available data for the variables under consideration.

No correction for multiple comparisons was applied, as these analyses were exploratory and hypothesis-generating; unadjusted *p* values are therefore reported.

A *p* value < 0.05 was considered statistically significant. Statistical analyses were performed using SPSS Statistics software (version 29.0.2.0; IBM Corp., Armonk, NY, USA).

## 3. Results

### 3.1. Patients and HCs

Of the 94 SSc patients, 79 (84.0%) were female. The median age was 59 (IQR 49–70) years, and the median disease duration was 8 (IQR 3–13) years. Sixty-four patients (68.1%) had limited cutaneous SSc, while 30 (31.9%) had diffuse cutaneous SSc. Interstitial lung disease was present in 60 patients (63.8%), and pulmonary hypertension, confirmed by right heart catheterisation, in 8 (8.5%). Details on demographic, clinical, and therapeutic characteristics of the SSc cohort are reported in Table 1.

Of the 50 HCs, 34 (68.0%) were female. The mean age was 47.1 (±14.6) years.

### 3.2. LS, LS ≥ 5.3 kPa and LS ≥ 7.5 kPa in SSc Patients and HCs

**Figure 1:**
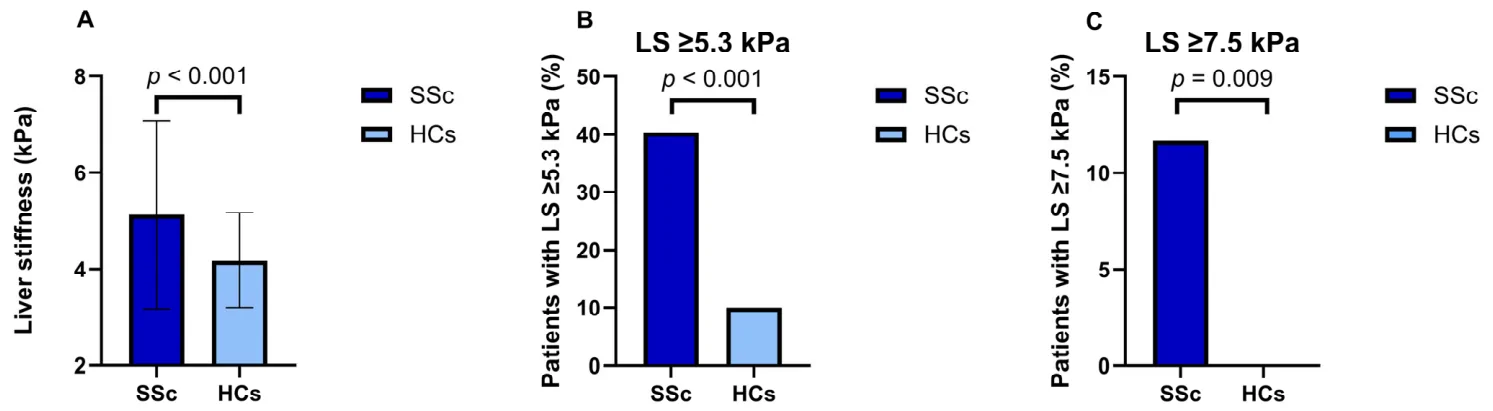
Comparison of liver stiffness measurements between systemic sclerosis (SSc) patients and healthy controls (HCs): (**A**) Liver stiffness (LS) values expressed in kPa. (**B**) Prevalence of LS ≥ 5.3 kPa. (**C**) Prevalence of LS ≥ 7.5 kPa.

In univariate analysis, LS was significantly higher in SSc patients compared with HCs (5.12 ± 1.95 kPa, median 4.70 [IQR 3.63–6.28] vs. 4.18 ± 0.98 kPa, median 4.00 [IQR 3.70–4.70], *p* < 0.001) (Figure 1). Multivariate analysis, adjusted for sex and age, confirmed higher LS values in SSc patients (β = 0.289, *p* = 0.001).

The prevalence of LS ≥ 5.3 kPa was significantly higher in SSc patients compared with HCs (38/94, 40.4% vs. 5/50, 10.0%; *p* < 0.001). In multivariate logistic regression analysis, adjusted for sex and age, LS ≥ 5.3 kPa was confirmed to be independently associated with SSc (adjOR 6.85, 95%CI 2.39 to 20.3, *p* = 0.001).

Similarly, the prevalence of LS ≥ 7.5 kPa was significantly higher in SSc patients than in HCs (11/94, 11.7% vs. 0/50, 0%; *p* = 0.009). Logistic regression was not performed since no HC had LS ≥ 7.5 kPa.

### 3.3. Factors Associated with Liver Stiffness Abnormalities

Higher LS values were significantly associated only with telangiectasias (5.5 ± 2.1 vs. 4.6 ± 1.6 kPa, *p* = 0.015) and serum GOT levels (ρ = 0.235, *p* = 0.023). In multivariate linear regression analysis, higher LS values remained independently associated with both telangiectasias (β = 0.222, *p* = 0.044) and GOT levels (β = 0.278, *p* = 0.012) (Supplementary Table S1a,b).

When considering LS ≥ 5.3, it was associated with ACA positivity (17/38, 44.7% vs. 13/56, 23.2%, *p* = 0.028) and higher serum GOT levels (22 [IQR 17–30] vs. 19 [IQR 17–23] U/L, *p* = 0.021) in univariate analysis. In multivariate logistic regression analysis, both ACA positivity (adjOR 4.09, 95% CI 1.29–12.96, *p* = 0.017) and GOT levels (adjOR 1.082, 95% CI 1.013–1.157, *p* = 0.020) remained independently associated with LS ≥ 5.3 kPa (Supplementary Table S2). No other demographic, clinical, serological, or treatment-related variables were significantly associated with LS ≥ 5.3 kPa.

Patients with LS ≥ 7.5 kPa more frequently had pulmonary hypertension (3/11, 27.3% vs. 5/83, 6.0%, *p* = 0.049), in particular pre-capillary pulmonary hypertension (3/11, 27.3% vs. 2/83, 2.4%, *p* = 0.011), and had higher GOT levels (24 [IQR 20–33] vs. 19 [IQR 16–26] U/L, *p* = 0.032) (Supplementary Table S3). No multivariate model was fitted for this outcome because of the small number of events.

## 4. Discussion

This exploratory cross-sectional study aimed to investigate the potential increase in LS in SSc patients and to identify factors associated with it. Liver involvement in SSc is not routinely evaluated by physicians beyond regular monitoring of hepatic biomarkers, primarily linked to the use of potentially hepatotoxic drugs, or for screening of co-existing autoimmune conditions like PBC or viral hepatitis [21]. For assessing LS, we chose TE because of its feasibility and reliability. Indeed, TE has proven to be a reliable non-invasive technique providing reproducible measurements under appropriate technical conditions and is considered a cost-effective tool for liver fibrosis assessment [22,23,24].

This study revealed that SSc patients had a significantly higher mean LS compared to HCs. We assessed LS both as a continuous variable and as a dichotomous variable, applying two different thresholds: LS ≥ 5.3 kPa and LS ≥ 7.5 kPa, recorded in around 40% and 12% of SSc patients, respectively. Previous TE studies have reported comparable proportions of patients above similar thresholds (ranging from 13.6% to 16.5%) [12,25]; however, some of these observations did not exclude subjects with pre-existing hepatic disease. In contrast, Cuomo et al., also using TE, reported a lower proportion (3.4%) comparable to that expected in the general population, although without a healthy control group for comparison [26]. Our findings are consistent with a higher prevalence of elevated liver stiffness in SSc. Liver stiffness in SSc has mostly been assessed as a dichotomous variable, whereas its evaluation as a continuous measure remains less common. The higher LS values observed in this cohort compared with HCs are compatible with hepatic involvement of the fibrotic process in SSc, although other mechanisms cannot be excluded. To minimise confounding, all patients were screened for concomitant viral hepatitis and autoimmune liver disease.

Among disease characteristics, neither disease duration (ρ = 0.194, *p* = 0.088) nor pulmonary function (FVC ρ = −0.073, *p* = 0.507; DLCO ρ = −0.084, *p* = 0.454) was associated with liver stiffness. Regarding laboratory parameters, GOT correlated with liver stiffness (ρ = 0.235, *p* = 0.023) and remained independently associated in multivariate analysis (β = 0.278, *p* = 0.012), whereas GPT did not reach significance (ρ = 0.183, *p* = 0.078). When the higher threshold of LS ≥ 7.5 kPa was applied, the only associated variables were pulmonary hypertension (*p* = 0.049), in particular pre-capillary pulmonary hypertension (*p* = 0.011), and GOT (*p* = 0.032), although multivariate analysis was not performed because of the small number of events.

GOT and GPT correlated with liver stiffness to a similar degree (ρ = 0.235 and ρ = 0.183), and only GOT remained significant in the multivariate model. This difference should be interpreted with caution: one coefficient fell just below and the other just above the conventional threshold, which in a cohort of this size does not indicate a different behaviour of the two enzymes. The study was not designed to detect associations of this magnitude, and the models included a fixed set of confounders that does not cover all potential determinants of liver stiffness, so residual confounding cannot be excluded. The single-centre design further limits the generalisability of these observations. From a biological standpoint, GOT is less liver-specific than GPT, being also expressed in skeletal and cardiac muscle, which is of particular relevance in systemic sclerosis, where subclinical muscle involvement is common. These findings should therefore be regarded as exploratory.

Two of the variables independently associated with liver stiffness, ACA positivity with LS ≥ 5.3 kPa (adjOR 4.09, 95% CI 1.29–12.96, *p* = 0.017) and telangiectasias with LS as a continuous variable (β = 0.222, *p* = 0.044), both belong to the vascular side of the disease rather than the fibrotic one. Whether liver stiffness in SSc has more to do with microvascular involvement than with fibrosis is something a cross-sectional study cannot establish and would require longitudinal data and a direct assessment of vascular involvement.

The associations observed with ACA positivity, telangiectasias, and GOT reached nominal significance only and would not survive correction for multiple comparisons; they may therefore represent chance findings and require confirmation in independent cohorts.

An alternative explanation for increased liver stiffness in SSc deserves consideration. Passive hepatic congestion secondary to right ventricular dysfunction is a recognised cause of increased liver stiffness in the absence of fibrosis. In our cohort, pulmonary hypertension was confirmed by right heart catheterisation in eight patients. Liver stiffness did not differ significantly between patients with and without pulmonary hypertension, but pulmonary hypertension—and particularly pre-capillary pulmonary hypertension—was more frequent among patients with LS ≥ 7.5 kPa. NT-proBNP did not correlate with liver stiffness. A contribution of hepatic congestion to the highest liver stiffness values cannot therefore be excluded, although the small number of affected patients precludes any firm conclusion. Systematic assessment of right ventricular function and of signs of hepatic congestion would be required to separate congestion from fibrosis as determinants of liver stiffness in SSc. Overall, these findings suggest that few clinical correlates of LS ≥ 7.5 kPa can be identified in SSc. Therefore, liver involvement may remain clinically silent and not predictable based on disease features alone, supporting the need for a broader and potentially systematic assessment of liver stiffness in SSc patients, even in the absence of overt risk factors.

Our study had several limitations. First, while TE is a widely used technique to investigate liver stiffness, its reliability specifically in patients with SSc has not been extensively investigated. Skin and subcutaneous fibrosis could theoretically affect the measurement, although TE was performed through the right intercostal spaces and LS values did not differ between the limited and diffuse cutaneous subsets (5.2 ± 2.0 vs. 5.0 ± 1.7 kPa, *p* = 0.968). Individual success rates and IQR/median ratios were not recorded and could therefore not be compared between groups. In our study, even when LS values ≥ 7.5 kPa were detected, liver biopsy was not proposed to patients. In the absence of suggestive antibodies or significant biochemical liver alterations, we did not consider it ethically justifiable to perform invasive procedures. Second, the cross-sectional design of our study did not permit serial LS measurements. Such longitudinal data would be valuable for monitoring patients, especially those with borderline values, to better understand the evolution of the condition. In addition, the limited number of patients with LS ≥ 7.5 kPa reduced the robustness of the analyses regarding this outcome. Third, the FibroScan^®^ 502 device used in this study was not equipped with the CAP software module, and concomitant liver ultrasound was not performed; hepatic steatosis could therefore not be assessed. As steatosis is a recognised determinant of LS, its contribution to our findings cannot be excluded, and the inclusion of patients with undiagnosed MASLD cannot be ruled out. Moreover, BMI and lipid profile were not available for HCs, so residual metabolic confounding in the comparison between the two groups cannot be excluded. Waist circumference, quantified alcohol intake, and anti-La antibodies were not available, and ALP was measured in only a minority of patients; all of these have been associated with increased liver stiffness in SSc in previous studies [12]. Right ventricular function and signs of hepatic congestion were not systematically assessed, so a contribution of passive hepatic congestion to the observed liver stiffness values cannot be excluded. Fourth, this was a single-centre study with restrictive exclusion criteria: patients with known liver disease, viral hepatitis, or AMA positivity were not enrolled, which by design excluded the forms of liver involvement most frequently reported in SSc and limits the external validity and representativeness of our findings. Consistently, the antibody distribution of our cohort, with ACA and anti-Scl-70 positivity at similar frequencies, differs from that of most European SSc cohorts, and no patient with SSc sine scleroderma was included; therefore, an unquantified selection at enrolment cannot be excluded. Fifth, the control group consisted of hospital healthcare workers, younger and less frequently female than the SSc patients and without available metabolic data; adjustment for age and sex cannot fully account for these differences, and a healthy worker effect cannot be excluded. Some clinical data were also incomplete: oesophageal involvement was assessed instrumentally only in symptomatic patients, lower gastrointestinal involvement was not systematically evaluated, and the extent of ILD on HRCT was not quantified.

Finally, this study was exploratory in nature: the sample size was not determined by a formal power calculation, the analyses of factors associated with liver stiffness were not pre-specified, and no correction for multiple comparisons was applied. These associations should therefore be regarded as hypothesis-generating. Multicentre studies with larger cohorts, pre-specified outcomes, and longitudinal follow-up of liver stiffness would be needed to test them formally.

In conclusion, our study suggests that patients with SSc exhibit increased liver stiffness compared with HCs, with LS ≥ 7.5 kPa recorded in 11.7% of our SSc cohort. TE may represent a useful non-invasive tool for detecting increased liver stiffness in SSc patients, potentially allowing for earlier diagnosis, even in asymptomatic cases or those without altered liver enzyme tests. Early detection may support closer monitoring and a more comprehensive assessment of liver involvement in SSc patients. These findings derive from a single centre and from a comparison with an unmatched control group. Further investigations involving larger patient cohorts, ideally with longitudinal evaluations, are needed to fully understand the precise burden of liver involvement in SSc.

## Figures and Tables

**Table 1:** Characteristics of SSc patients (*n* = 94).

Variable	Value
**Demographic characteristics**	
Female sex, *n* (%)	79 (84.0%)
Male sex, *n* (%)	15 (16.0%)
Age, years, median (IQR)	59 (49–70)
BMI, kg/m^2^, mean (±SD)	23.5 (±3.9)
**Comorbidities**	
Arterial hypertension, *n* (%)	21 (24.1%)
Diabetes, *n* (%)	3 (3.4%)
Total cholesterol, mg/dL, median (IQR)	197 (172–221)
**Disease characteristics and serological profile**	
Disease duration, years, median (IQR)	8 (3–13)
ANA positivity, *n* (%)	94 (100%)
ACA positivity, *n* (%)	30 (31.9%)
Anti-Scl-70 positivity, *n* (%)	26 (27.7%)
Limited cutaneous SSc, *n* (%)	64 (68.1%)
Diffuse cutaneous SSc, *n* (%)	30 (31.9%)
**Clinical manifestations**	
Raynaud’s phenomenon, *n* (%)	90 (95.7%)
History of digital ulcers, *n* (%)	47 (50.0%)
Calcinosis, *n* (%)	19 (20.2%)
Telangiectasias, *n* (%)	55 (58.5%)
Oesophageal involvement, *n* (%)	55 (58.5%)
Interstitial lung disease, *n* (%)	60 (63.8%)
Pulmonary hypertension, *n* (%)	8 (8.5%)
Pre-capillary pulmonary hypertension, *n* (%)	5 (5.3%)
Post-capillary pulmonary hypertension, *n* (%)	3 (3.2%)
**Therapy**	
Immunosuppressive therapy, *n* (%)	42 (44.7%)
Antifibrotic, *n* (%)	3 (3.2%)
Methotrexate, *n* (%)	8 (9.2%)
History of corticosteroid use, *n* (%)	69 (73.4%)
Endothelin receptor antagonist therapy, *n* (%)	27 (28.7%)
History of IV prostanoids, *n* (%)	68 (72.3%)
Phosphodiesterase-5 inhibitors, *n* (%)	24 (25.5%)

Continuous variables are presented as median (IQR) or mean (SD), as indicated; categorical variables are presented as number (%). Abbreviations: SSc, systemic sclerosis; BMI, body mass index; ANAs, antinuclear antibodies; ACAs, anti-centromere antibodies; IV, intravenous.

## Data Availability

The data presented in this study are available upon reasonable request from the corresponding author. The data are not publicly available due to ethical and privacy restrictions related to patient data.

## Supplementary Materials

The following supporting information can be downloaded at: https://www.mdpi.com/article/10.3390/jcm15218593/s1, Table S1a: Factors (dichotomous variables) associated with liver stiffness (continuous variable) in SSc patients; Table S1b: Factors (continuous variables) associated with liver stiffness (continuous variable) in SSc patients; Table S2: Factors associated with LS ≥ 5.3 kPa in SSc patients; Table S3: Factors associated with LS ≥ 7.5 kPa in SSc patients.
