# Supplementary material for: Increased Liver Stiffness in Systemic Sclerosis: A Single-Centre Transient Elastography Study
Source: J Clin Med. 2026 Nov 4;15(21):8593. doi: 10.3390/jcm15218593 (PMC13763636; doi:10.3390/jcm15218593)
Supplement: Supplementary file 1 [file jcm-15-08593-s001.zip › jcm-4576444-supplementary.pdf]

Supplementary Table S1a. Factors (dichotomous variables) associated with liver stiffness (continuous variable) in SSc patients.

Variable	Univariate analysis		Multivariate analysis	
	**Mean LS (SD), kPa**	**p-value**	**β coefficient**	**p-value**
Demographics				
Female vs male sex	5.2 (±2.0) vs 4.8 (±1.3)	0.729		
Comorbidities				
Arterial hypertension + vs −	5.2 (±1.9) vs 5.0 (±2.0)	0.620		
Diabetes + vs −	5.7 (±1.7) vs 5.0 (±2.0)	0.351		
Disease characteristics and serological profile				
ACA + vs −	5.6 (±2.1) vs 4.9 (±1.9)	0.187		
Anti-Scl-70 + vs −	4.8 (±1.6) vs 5.3 (±2.0)	0.407		
dcSSc vs lcSSc	5.0 (±1.7) vs 5.2 (±2.0)	0.968		
Clinical manifestations				
Raynaud's phenomenon + vs −	5.1 (±1.9) vs 4.9 (±2.3)	0.822		
History of digital ulcers + vs −	5.1 (±1.8) vs 5.1 (±2.1)	0.642		
Calcinosis + vs −	5.3 (±2.1) vs 5.1 (±1.9)	0.745		
Telangiectasias + vs −	5.5 (±2.1) vs 4.6 (±1.6)	**0.015**	0.222	**0.044**
Oesophageal involvement + vs −	5.2 (±1.8) vs 5.0 (±2.1)	0.363		
Interstitial lung disease + vs −	5.1 (±2.0) vs 5.1 (±1.9)	0.798		
Pulmonary hypertension + vs −	6.1 (±2.6) vs 5.0 (±1.9)	0.244		
Pre-capillary PH + vs −	7.1 (±2.9) vs 5.0 (±1.8)	0.081		
Post-capillary PH + vs −	4.5 (±1.1) vs 5.1 (±2.0)	0.706		
Therapy				
Immunosuppressive or antifibrotic + vs −	5.2 (±2.0) vs 4.9 (±1.7)	0.544		
History of corticosteroid use + vs −	5.0 (±1.7) vs 5.5 (±2.5)	0.676		
Endothelin receptor antagonist + vs −	5.2 (±2.0) vs 5.1 (±1.9)	0.834		
Methotrexate + vs −	5.5 (±3.0) vs 5.0 (±1.9)	0.959		
History of IV prostanoids + vs −	5.2 (±2.0) vs 4.9 (±1.7)	0.622		

Abbreviations — SSc, systemic sclerosis; LS, liver stiffness; ACA, anti-centromere antibodies; dcSSc, diffuse cutaneous systemic sclerosis; lcSSc, limited cutaneous systemic sclerosis; PH, pulmonary hypertension; IV, intravenous. Missing data were not imputed. Each analysis included only participants with available data for the variables under consideration.

Supplementary Table S1b. Factors (continuous variables) associated with liver stiffness (continuous variable) in SSc patients.

Variable	Univariate analysis		Multivariate analysis	
	Spearman's coefficient	p-value	β coefficient	p-value
Demographics				
Age (years)	-0.171	0.100		
Disease duration (years)	0.194	0.088		
BMI (kg/m²)	-0.002	0.988		
Clinical manifestations				
FVC, % predicted	-0.073	0.507		
DLCO, % predicted	-0.084	0.454		
Laboratory parameters				
GOT (U/L)	0.235	**0.023**	0.278	**0.012**
GPT (U/L)	0.183	0.078		
GGT (U/L)	0.044	0.674		
ALP (U/L)	0.392	0.166		
NT-proBNP (pg/mL)	-0.132	0.271		
Total cholesterol (mg/dL)	0.035	0.737		

Abbreviations — SSc, systemic sclerosis; BMI, body mass index; FVC, forced vital capacity; DLCO, diffusing capacity of the lung for carbon monoxide; GOT, glutamic-oxaloacetic transaminase; GPT, glutamic-pyruvic transaminase; GGT, gamma-glutamyl transferase; ALP, alkaline phosphatase; NT-proBNP, N-terminal pro-B-type natriuretic peptide.

Missing data were not imputed. Each analysis included only participants with available data for the variables under consideration.

Supplementary Table S2. Factors associated with LS ≥5.3 kPa in SSc patients.

Variable	Univariate analysis			Multivariate analysis	
	LS ≥5.3 kPa (n = 38)	LS <5.3 kPa (n = 56)	p-value	adjOR (95% CI)	p-value
Demographics					
Female sex, n (%)	34 (89.5%)	45 (80.4%)	0.268		
Age, years, median (IQR)	58 (49-69)	59 (51-70)	0.525		
BMI, kg/m²	23.8 (3.8)	23.4 (3.4)	0.633		
Comorbidities					
Arterial hypertension, n (%)	7 (20.6%)	14 (26.4%)	0.535		
Diabetes, n (%)	1 (2.9%)	2 (3.8%)	1.000		
Disease characteristics and serological profile					
Disease duration, years, median (IQR)	8 (4-16)	8 (3-12)	0.316		
ACA positivity, n (%)	17 (44.7%)	13 (23.2%)	**0.028**	4.091 (1.291-12.962)	**0.017**
Anti-Scl-70 positivity, n (%)	8 (21.1%)	18 (32.1%)	0.238		
dcSSc, n (%)	11 (28.9%)	19 (33.9%)	0.611		
Clinical manifestations					
Raynaud's phenomenon, n (%)	36 (94.7%)	54 (96.4%)	1.000		
History of digital ulcers, n (%)	19 (50.0%)	28 (50.0%)	1.000		
Calcinosis, n (%)	7 (18.4%)	12 (21.4%)	0.722		
Telangiectasias, n (%)	26 (68.4%)	29 (51.8%)	0.130		
Oesophageal involvement, n (%)	24 (63.2%)	31 (55.4%)	0.451		
Interstitial lung disease, n (%)	24 (63.2%)	36 (64.3%)	0.911		
FVC, % predicted, median (IQR)	104 (79-120)	103 (87-129)	0.608		
DLCO, % predicted	64.2 (19.6)	65.7 (20.2)	0.731		
Pulmonary hypertension, n (%)	4 (10.5%)	4 (7.1%)	0.711		
Pre-capillary PH, n (%)	3 (7.9%)	2 (3.6%)	0.391		
Post-capillary PH, n (%)	1 (2.6%)	2 (3.6%)	1.000		
Laboratory parameters					
GOT (U/L), median (IQR)	22 (17-30)	19 (17-23)	**0.021**	1.082 (1.013-1.157)	**0.020**
GPT (U/L), median (IQR)	17 (11-26)	16 (12-21)	0.443		
GGT (U/L), median (IQR)	17 (13-30)	19 (16-34)	0.324		
ALP (U/L), median (IQR)	73 (63-93)	59 (42-74)	0.155		
Total cholesterol, mg/dL, median (IQR)	199 (173-221)	197 (167-221)	0.661		
NT-proBNP, pg/mL, median (IQR)	120 (98-278)	203 (89-493)	0.533		
Therapy					
Immunosuppressive or antifibrotic, n (%)	19 (50.0%)	26 (46.4%)	0.865		
History of corticosteroid use, n (%)	27 (71.1%)	42 (75.0%)	0.818		
Endothelin receptor antagonist, n (%)	10 (26.3%)	17 (30.4%)	0.671		
Methotrexate, n (%)	4 (11.8%)	4 (7.5%)	0.507		

History of IV prostanoids, n (%)	28 (73.7%)	40 (71.4%)	0.817

Continuous variables are presented as median (IQR), except BMI and DLCO, which are presented as mean (SD); categorical variables are presented as number (%).
Abbreviations — SSc, systemic sclerosis; LS, liver stiffness; BMI, body mass index; ACA, anti-centromere antibodies; dcSSc, diffuse cutaneous systemic sclerosis; FVC, forced vital capacity; DLCO, diffusing capacity of the lung for carbon monoxide; PH, pulmonary hypertension; GOT, glutamic-oxaloacetic transaminase; GPT, glutamic-pyruvic transaminase; GGT, gamma-glutamyl transferase; ALP, alkaline phosphatase; NT-proBNP, N-terminal pro-B-type natriuretic peptide; IV, intravenous.
Missing data were not imputed. Each analysis included only participants with available data for the variables under consideration.

Supplementary Table S3. Factors associated with LS ≥7.5 kPa in SSc patients.

Variable	LS ≥7.5 kPa (n = 11)	LS <7.5 kPa (n = 83)	p-value
Demographics			
Female sex, n (%)	11 (100%)	68 (81.9%)	0.202
Age, years, median (IQR)	51 (49-69)	59 (49-70)	0.560
BMI, kg/m²	24.3 (4.2)	23.4 (3.8)	0.485
Comorbidities			
Arterial hypertension, n (%)	2 (20.0%)	19 (24.7%)	1.000
Diabetes, n (%)	1 (10.0%)	2 (2.6%)	0.307
Disease characteristics and serological profile			
Disease duration, years, median (IQR)	10 (7-20)	8 (3-13)	0.261
ACA positivity, n (%)	4 (36.4%)	26 (31.3%)	0.740
Anti-Scl-70 positivity, n (%)	3 (27.3%)	23 (27.7%)	1.000
dcSSc, n (%)	5 (45.5%)	25 (30.1%)	0.305
Clinical manifestations			
Raynaud's phenomenon, n (%)	11 (100%)	79 (95.2%)	1.000
History of digital ulcers, n *(%)*	6 (54.5%)	41 (49.4%)	0.748
Calcinosis, n (%)	2 (18.2%)	17 (20.5%)	1.000
Telangiectasias, n (%)	9 (81.8%)	46 (55.4%)	0.115
Oesophageal involvement, n (%)	8 (72.7%)	47 (56.6%)	0.352
Interstitial lung disease, n (%)	7 (63.6%)	53 (63.9%)	0.989
FVC, % predicted, median (IQR)	109 (79-128)	103 (86-120)	0.591
DLCO, % predicted	62.4 (16.9)	65.5 (20.3)	0.672
Pulmonary hypertension, n (%)	3 (27.3%)	5 (6.0%)	**0.049**
Pre-capillary PH, n (%)	3 (27.3%)	2 (2.4%)	**0.011**
Post-capillary PH, n (%)	0 (0%)	3 (3.6%)	1.000
Laboratory parameters			
GOT, (U/L), median (IQR)	24 (20-33)	19 (16-26)	**0.032**
GPT, (U/L), median (IQR)	22 (14-48)	16 (11-22)	0.098
GGT, (U/L), median (IQR)	20 (13-100)	19 (15-30)	0.584
ALP, (U/L), median (IQR)	127 (66-187)	63 (49-81)	0.144
Total cholesterol, mg/dL, median (IQR)	182 (166-210)	197 (173-221)	0.315
NT-proBNP, pg/mL, median (IQR)	113 (91-597)	198 (98-433)	0.782
Therapy			
Immunosuppressive or antifibrotic therapy, n (%)	6 (54.5%)	39 (47.0%)	0.692
History of corticosteroid use, n (%)	7 (63.6%)	62 (74.7%)	0.492
Endothelin receptor antagonist therapy, n (%)	4 (36.4%)	23 (27.7%)	0.724
Methotrexate, n (%)	1 (10.0%)	7 (9.1%)	1.000
History of IV prostanoids, n (%)	9 (81.8%)	59 (71.1%)	0.411

Continuous variables are presented as median (IQR), except BMI and DLCO, which are presented as mean (SD). Abbreviations — SSc, systemic sclerosis; BMI, body mass index; ACA, anti-centromere antibodies; dcSSc, diffuse cutaneous systemic sclerosis; FVC, forced vital capacity; DLCO, diffusing capacity of the lung for carbon monoxide; PH, pulmonary hypertension; GOT, glutamic-oxaloacetic transaminase; GPT, glutamic-pyruvic transaminase; GGT, gamma-glutamyl transferase; ALP, alkaline phosphatase; NT-proBNP, N-terminal pro-B-type natriuretic peptide; IV, intravenous. Missing data were not imputed. Each analysis included only participants with available data for the variables under consideration.
